# Prevention of Gastric Cancer

**DOI:** 10.3390/medicina62040660

**Published:** 2026-03-31

**Authors:** Simona-Maria Bățagă, Paul Grama, Monica Pantea, Sergiu Frandeș, Naomi-Adina Ciurea

**Affiliations:** 1 M3 Department, Discipline of Internal Medicine 1, George Emil Palade University of Medicine, Pharmacy, Science, and Technology of Târgu Mureș, 540139 Târgu Mureș, Romania; 2 Department of Gastroenterology, Târgu Mures Emergency Clinical County Hospital, 540136 Târgu Mureș, Romania; 3 M3 Department, Discipline of Internal Medicine 2, George Emil Palade University of Medicine, Pharmacy, Science, and Technology of Târgu Mureș, 540139 Târgu Mureș, Romania

**Keywords:** gastric cancer, preneoplastic lesions, helicobacter pylori, screening, prevention

## Abstract

Gastric cancer (GC) is the fifth most common malignancy worldwide, with 968,784 new cases reported in 2022. Since 1975, when stomach cancer was the most common cancer, its incidence has declined in many regions. This decline can be attributed to improved food preservation and recognition of *Helicobacter pylori* (*H. pylori*) as a group 1 carcinogen in intestinal-type GC. The aim of this review was to summarize current strategies for primary and secondary prevention of GC, with an emphasis on *H. pylori* management, dietary factors, novel biomarkers, and screening approaches relevant in Europe. Papers from large databases, namely Web of Science, Scopus, and PubMed/MEDLINE, were selected (reviews, guidelines, and peer-reviewed studies) from about the last 1–5 years. The research was conducted using the keywords gastric cancer, prevention, primary prevention, secondary prevention, and endoscopy guidelines on prevention of gastric cancer. Primary prevention of GC is directed at screening for *H. pylori* and dietary changes. Secondary prevention is supported by traditional tumor markers, pepsinogen-based serological biopsy, newer blood-based markers, and major technological progress in endoscopy. High-definition endoscopy, magnification, virtual chromoendoscopy, and artificial intelligence have improved the detection of preneoplastic lesions and early cancer, while advanced therapeutic techniques such as endoscopic mucosal resection and endoscopic submucosal dissection permit organ-sparing treatment. Large projects, including GISTAR, EUROHELICAN, TOGAS, and EUCanScreen, are expected to clarify optimal screening strategies. Although GC incidence has declined, it remains a common and significant malignancy. Understanding the facets of primary and secondary prevention of GC will lead to a reduction in the burden of this disease.

## 1. Introduction

In 1975, stomach cancer was the most common neoplasm. As of 2022, gastric cancer (GC) ranks as the fifth most common malignancy worldwide, with 968,784 new cases, and the fifth-leading cause of cancer-related deaths, accounting for 7.7% of all cancers worldwide [1]. Despite this decrease from 1975 to 2022, prediction models have shown that the annual number of new GC cases and GC-related deaths will increase to 1.77 million and 1.27 million, respectively, by 2040 [2]. A recent study estimated that among people born from 2008 to 2017, 15.6 million (95% uncertainty interval 14.0–17.3 million) lifetime GC cases are expected, with most attributable to Helicobacter pylori (*H. pylori*) infection [3]. Clearly, GC still contributes to a significant disease burden on individuals, healthcare systems, and the world economy.

Several factors have contributed to the initial decline in GC incidence. In 1982, *H. pylori* was discovered, and since then, several treatments have been investigated to help with the eradication of the infection. Additionally, the introduction of food preservation by freezing in the 1970s has decreased the number of gastric-related infections that can lead to GC. The creation of the management of epithelial precancerous conditions and early neoplasia of the stomach (MAPS) guidelines detailing the follow-up of preneoplastic lesions, and the development of endoscopy to detect small lesions have also contributed to the decline in GC incidence. Even though the incidence has decreased, GC remains one of the most important causes of cancer mortality worldwide. It is most often diagnosed at an advanced, incurable stage because early GC (EGC) that is potentially curable presents asymptomatically. Patients typically present only when symptoms are more pronounced, leading to delayed diagnosis. In Western countries, including Europe and the United States, the diagnosis is typically made after the cancer has invaded the muscularis propria, contributing to a 5-year survival rate below 20%. However, patients diagnosed with EGC have a significantly better prognosis, with a 5-year survival rate approaching 90% [4].

**Figure 1:**
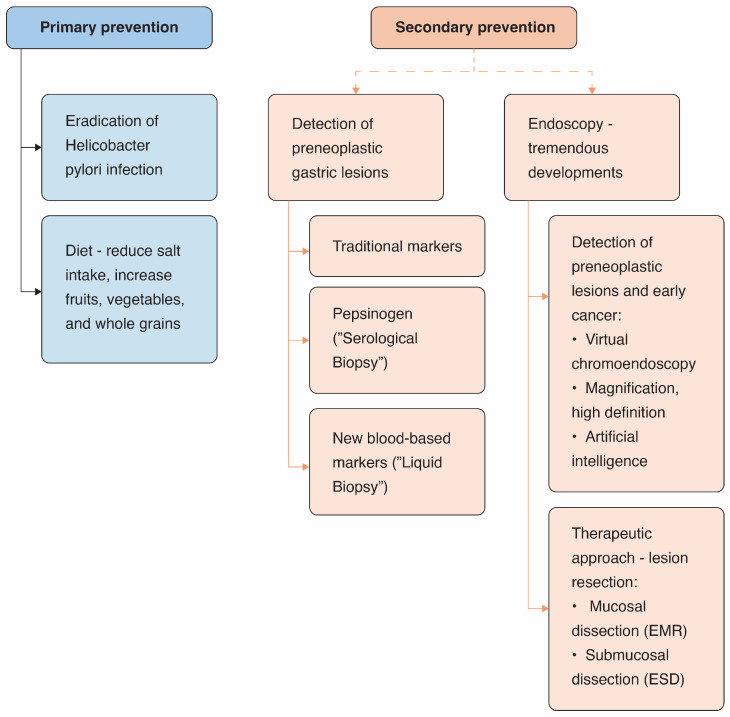
Gastric cancer prevention.

The prevention of GC is classified into two levels. Primary prevention focuses on the eradication of *H. pylori* infection and the promotion of a healthy diet. Secondary prevention targets the detection of preneoplastic gastric lesions and EGC (Figure 1). This review aimed to summarize primary and secondary prevention strategies for GC.

This article is a narrative review. The literature search was performed in PubMed/MEDLINE, Scopus, and Web of Science using combinations of the following terms: gastric cancer, prevention, primary prevention, secondary prevention, and endoscopy guidelines on prevention of gastric cancer. We considered articles published in English between 2020 and 2025. Priority was given to international guidelines, meta-analyses, randomized and prospective studies, and high-impact observational studies relevant to primary and secondary prevention of gastric cancer. Conference abstracts, non-English publications, and studies not directly related to prevention or early detection were not prioritized. The evidence was synthesized narratively, with emphasis on clinically relevant data applicable to European practice. When overlapping or discordant guidelines or meta-analyses were identified, preference was given to the most recent and methodologically robust source. When multiple quantitative estimates were available, we prioritized those derived from higher-level evidence and from studies using the most clinically comparable endpoint definitions.

## 2. Primary Prevention of Gastric Cancer

### 2.1. H. pylori Infection

Since the discovery of *H. pylori* in 1982 by Warren and Marshall [5], studies have demonstrated that *H. pylori* contributes significantly to the pathogenesis of intestinal-type GC. Likewise, the prevention of *H. pylori* infection contributes significantly to preventing GC. The role of *H. pylori* in carcinogenesis has been determined through the sequence of metaplasia, then dysplasia, and finally GC. This model was proposed in 1988 by Correa [6,7] and revealed the malignant transformation from *H. pylori* infection to GC as the Correa cascade. In 1994, *H. pylori* was classified as a group 1 carcinogen by the International Agency for Research on Cancer.

### 2.2. Other Risk Factors

An important step in the prevention of GC is the preservation of food by freezing. Before this advancement, food was preserved by salt, smoking, nitrosamines, and other nitroso compounds, which promote gastric carcinogenesis [8]. Other risk factors for GC are smoking and alcohol consumption. A meta-analysis of 42 cohort studies, case cohorts, and case-controlled cohorts conducted in Asia, Europe, and the United States identified a relative risk of GC of 1.53 (1.42–1.65) among individuals who smoke [9]. Alcohol in high doses (more than 4 drinks per day) is correlated with an increased incidence of GC [10]. Finally, obesity and a diet rich in red meat increases the risk of GC [11].

Evidence regarding fruit intake and gastric cancer prevention is suggestive but not definitive. Prospective cohort data support a possible inverse association, although the magnitude of benefit appears modest and may vary by population. In the Japanese Public Health Center-based prospective study, fruit intake showed only a limited inverse association with gastric cancer risk, indicating that findings from pooled international analyses should be interpreted cautiously when applied to local prevention strategies. Therefore, increased fruit consumption can be presented as part of an overall healthy dietary pattern, but not as a stand-alone validated preventive intervention for gastric cancer [12].

### 2.3. Dietary Modulation of Gastric Carcinogenesis in the Context of H. pylori

Diet acts as a co-determinant of the gastric carcinogenic cascade by amplifying or attenuating the inflammatory–metaplastic trajectory initiated by *H. pylori*. Mechanistic and experimental studies suggest that high salt intake may impair the gastric mucosal barrier; enhance *H. pylori* virulence, including CagA-related effects; and promote oxidative and nitrosative stress, while epidemiologic studies in humans support an association between high-salt dietary patterns and increased gastric cancer risk. Recent microbiome-focused analyses also suggest that *H. pylori* and dietary patterns shape the gastric microbial ecosystem through a loss of diversity that accompanies the progression along the Correa pathway. However, this cascade can be partially reversed following eradication of *H. pylori* and dietary improvement [13].

The most compelling interventional evidence originates from the long-term Linqu randomized trial in a high-risk Chinese population (*n* = 3365). After combining *H. pylori* eradication and nutrition interventions (vitamins C and E, selenium, and garlic), reductions in GC incidence and mortality were observed over more than two decades of follow-up [14]. Although eradication accounted for the largest absolute risk reduction, nutritional optimization produced an additional, independent benefit, reinforcing diet as a risk-modifying co-determinant after eradication.

Cohort studies and umbrella reviews consistently show that plant-forward diets, particularly those rich in fruits; vegetables, specifically allium (onions and garlic) and brassica (cruciferous) vegetables; and naturally antioxidative foods, are associated with a lower GC risk [15], whereas a high intake of salt, smoked or pickled foods, and processed meats is associated with elevated risk. In addition to salt-related mucosal injury and potentiation of *H. pylori* virulence, nitroso compounds, derived from preserved meats, smoked foods, and endogenous nitrosation, may also contribute to gastric carcinogenesis. From a practical perspective, daily preventive measures include reducing processed and heavily preserved foods, limiting smoked and charred meat intake, favoring refrigeration over salt-based preservation, and increasing fresh fruit and vegetable consumption [16,17].

These data support a two-pillar prevention model: eradication to interrupt the upstream driver(s) of carcinogenesis, and dietary risk modification to attenuate the residual inflammatory field that persists after infection clearance. Dietary modification is not a substitute for eradication but a practical adjunct, particularly in regions with moderate incidence rates, where counseling can be integrated into screening/eradication protocols at minimal cost [18]. In Romania, where *H. pylori* prevalence is substantial and diagnosis often occurs in later disease stages, structured post-eradication counseling, for example, limiting salt intake to <5 g/day in line with public health recommendations [17], consuming ≥5 daily servings of fruits and vegetables, and routinely including allium and brassica vegetables, could convert a one-time medical intervention into sustained long-term prevention [19].

### 2.4. Population-Based Screening and Test-And-Treat Models for H. pylori Eradication

Preneoplastic gastric lesions include atrophic gastritis, intestinal metaplasia, and dysplasia. The greatest preventive effect is achieved when *H. pylori* infection is identified and treated before these precancerous changes become established. Follow-up of the patients with precancerous lesions should be performed according to MAPS III ESGE Guidelines [20].

Several countries in East Asia with a high incidence of gastric cancer have already implemented this strategy. Japan was the first nation to incorporate *H. pylori* eradication into a systematic GC prevention policy [21]. The national health insurance system in Japan expanded coverage to include *H. pylori* eradication therapy, effectively extending eligibility for treatment for most infected adults [22]. Since February 2013, *H. pylori* eradication has been implemented in Japan through organized screening programs (test-and-treat strategy) to decrease GC incidence through the detection and elimination of *H. pylori*.

South Korea integrated eradication into its national GC screening infrastructure to reach more than 70% of the eligible adult population [23]. Taiwan has implemented structured screening–eradication models within targeted high-risk regions, and China has transitioned from pilot programs to wide-scale public health initiatives [24]. A landmark cluster-randomized trial in China involving more than 180,000 adults with a follow-up of 11 years demonstrated a statistically significant reduction in incident GC among those who achieved successful eradication. This study confirmed the long-term preventive outcomes of community-based *H. pylori* eradication [25].

Several regions outside of East Asia have initiated similar pilot or hybrid implementation models [26]. Hong Kong and Singapore are actively evaluating feasibility frameworks, and structured initiatives such as TOGAS and EUROHELICAN in Europe have advanced conversations surrounding the policy logistics despite the absence of a national program [27]. European programs are currently hindered by implementation constraints: antimicrobial resistance of *H. pylori*, workforce infrastructure for the test-and-treat strategy, and uncertainties regarding cost-effectiveness thresholds [28]. Nevertheless, contemporary modeling indicates that mass or targeted screening is cost-effective in regions with a moderate or higher disease burden [29].

Romania may be considered a setting with a potentially relevant gastric cancer burden, in line with patterns observed in Central and Eastern Europe [30]; however, the lack of comprehensive national data on incidence, mortality, and stage distribution precludes a definitive classification [31,32]. In this context, Romania could represent a potential candidate for structured screening strategies; nevertheless, the feasibility of such programs requires further evaluation, including endoscopic capacity, antimicrobial resistance patterns, and cost-effectiveness considerations [33].

However, the European Union has not yet issued binding recommendations for organized *H. pylori* screening. Currently, active clinical trials and implementation studies (including those registered across European platforms) reflect a growing shift from concept to feasibility [34].

When viewed from an international collective, a directional trend from symptomatic treatment toward the structured identification and eradication of *H. pylori* infection as a cancer preventative measure is evident. Nations that have already implemented population-level screening provide a proof of concept for epidemiologic and logistic feasibility. Currently, countries in the pilot phase are adapting these models to infrastructural and antimicrobial-resistance constraints. For regions with a potentially relevant gastric cancer burden, the translational opportunity lies in adapting rather than reinventing validated prevention frameworks. *H. pylori* eradication must be identified as a gastroenterology intervention, as well as an upstream cancer prevention strategy aligned with global health policy evolution.

### 2.5. H. pylori Eradication as a Primary Prevention Strategy for GC

Gastric carcinogenesis is a multifactorial and stepwise process initiated by chronic mucosal inflammation and oxidative injury, leading to progressive atrophy, intestinal metaplasia, and dysplasia before malignant transformation [35]. *H. pylori* infection is recognized as the predominant modifiable trigger of this cascade [36], accounting for more than 70% of non-cardia GC cases worldwide [37]. The eradication of *H. pylori* represents the most direct and biologically grounded intervention to interrupt this pathogenic cascade [38]. When performed prior to precancerous initiation, bacterial clearance reverts the inflammatory microenvironment, reduces reactive oxygen species, restores epithelial integrity, and significantly decreases the probability of neoplastic progression [39].

A recent large-scale prospective study has provided compelling confirmation of this preventive effect at the population level. The study enrolled over 180,000 adults in a randomized, community-based trial with a median follow-up of 11.8 years. Eradication therapy significantly reduced GC incidence compared with placebo [hazard ratio: 0.86, 95% confidence interval (CI): 0.74–0.99], with a greater benefit in those achieving successful bacterial clearance (hazard ratio: 0.81; 95%CI: 0.69–0.96) [33]. No major adverse events were observed, supporting the feasibility and safety of implementing *H. pylori* eradication on a population scale. These findings consolidated the concept that structured, population-level eradication can modify gastric carcinogenesis beyond the scope of individual therapy.

The Taipei Global Consensus II on *H. pylori* Management formalized the paradigm shift from symptom-driven treatment to systematic infection control to prevent cancer [27]. The consensus recommended eradication therapy to all adults infected with *H. pylori* regardless of symptom status through the test-and-treat strategy. This consensus marked a substantial departure from reserving therapy for patients with dyspepsia or who were high-risk patients. By treating *H. pylori* as an oncogenic agent rather than a commensal pathogen, the consensus aligned with other infection-based cancer prevention measures, such as vaccination against oncogenic viruses.

Implementation science and cost-effectiveness analyses reinforced the relevance of this approach to public health. Modeling studies demonstrated that population-wide eradication programs are cost-effective in regions with moderate-to-high GC incidence when noninvasive testing and well-tolerated treatment regimens were employed [28]. This evidence suggests that countries with moderate incidence may derive a substantial benefit from organized screening and eradication initiatives, bridging preventive oncology with infectious-disease-control policies.

## 3. Secondary Prevention of Gastric Cancer

### 3.1. Traditional Markers

Tumor markers are routinely used for the early detection of GC. Tumor markers include carcinoembryonic antigen (CEA), carbohydrate antigen (CA) 72-4, CA 19-9, CA 15-3, and CA 125 to monitor therapy and predict the prognosis of GC. These markers may be elevated in GC, but they are neither sensitive nor specific to GC [40]. Several groups have investigated the combination of these markers or their combination with cytokines to increase the sensitivity and specificity. Currently, a combination of serum CEA and CA 19–9, or a combination of CEA, CA 125, and CA 19-9 has a higher specificity and sensitivity, respectively, than CEA alone [41]. Another study demonstrated that the combination of CEA, CA 72-4, tumor necrosis factor α, interleukin 6, and interleukin 8 could detect EGC [42].

### 3.2. Serological Biopsy: Markers for Atrophy of the Stomach Mucosa

Serological biopsy was coined by Samloff [43] to refer to the noninvasive diagnosis of precancerous conditions (*H. pylori* gastritis or atrophic gastritis) to identify patients with a high cancer risk who need endoscopy and cancer surveillance regardless of experiencing symptoms. Pepsinogens (PGs) are markers for atrophy, which is similar to intestinal metaplasia in the Correa cascade. Atrophy and intestinal metaplasia induce intestinal-type GC and are correlated with *H. pylori* infection.

There are two isozymogens, PGI that is produced only in the gastric corpus of the stomach, and PGII that is produced throughout the stomach and in the duodenal Brunner glands. The best marker is the ratio of PGI and PGII; however, recently, this marker alone has demonstrated low specificity [44,45]. Combining the PGI:PGII ratio and *H. pylori* antibodies has been used in the ABC method. Patients can be classified into four groups using *H. pylori* status and PG levels: (1) Group A, *H. pylori*-negative, PG-negative; (2) Group B, *H. pylori*-positive, PG-negative; (3) Group C, *H. pylori*-positive, PG-positive; and (4) Group D, *H. pylori*-negative, PG-positive (Table 1) [46].

The ABC method allows risk stratification as follows. Individuals in group A with a healthy gastric mucosa have a very low risk of gastric disease. Group B has an increased risk of peptic ulcer. Individuals in group C have a higher risk of diseases related to gastric mucosal atrophy, such as GC, gastric adenoma, and hyperplastic polyps. Individuals in group D with advanced atrophy are at particularly high risk of developing GC. The ABC method has been used to screen large populations with good results.

Another marker used in combination with PG is gastrin-17. Gastrin-17 characterizes atrophy in the antral part of the stomach because it is secreted exclusively by the G cells, leading to a high specificity for atrophic gastritis. Although PG has been used for many years in the detection of gastric atrophy, recent studies have confirmed its importance [46,47]. The 2025 European Society of Gastrointestinal Endoscopy (ESGE) guidelines, MAPS III, recommend serum pepsinogen for identifying individuals at increased risk of precancerous gastric conditions who should undergo endoscopic evaluation. Patients with low PGI levels or a low PGI/PGII ratio, particularly in the absence of detectable *H. pylori* antibodies, should undergo endoscopic screening or surveillance [20]. Other promising markers under study for the detection of atrophic gastritis and EGC are the hormone ghrelin and the trefoil factor.

### 3.3. Liquid Biopsy

Liquid biopsies are blood samples or other biofluids (saliva and urine) that are used for the analysis of cancer cells or cancer cell-derived molecules [48]. The most common liquid biopsy components are circulating tumor cells; various types of circulating cell-free RNA, such as methylation markers; cell-free mRNA; microRNA (miRNA); long noncoding RNA; and other RNA species, including characteristic miRNA fingerprints. Another key component is circulating tumor DNA, which is the fraction of cell-free DNA originating from primary tumors, metastases, and circulating tumor cells.

Extracellular vesicles, including exosomes, microvesicles, and apoptotic bodies, also play an important role as carriers of tumor-derived nucleic acids and proteins. Extracellular vesicles are an alternative source of cancer-derived biomarkers for liquid biopsies used for recurrence monitoring and prognostic and predictive biomarkers. One commercially available molecular blood test is GASTROClear, which uses a 12-miRNA serum biomarker panel. In the validation study by So et al. conducted in a high-risk population, this panel showed sensitivity approaching 90% for gastric cancer detection; however, diagnostic performance may vary according to disease stage and population characteristics [49].

## 4. New Methods of Prevention

### 4.1. Point-of-Care Testing Devices

Point of care refers to medical diagnostic testing that is performed without the need for a centralized laboratory facility, giving patients rapid results. Biological samples such as blood, saliva, urine, and breath can be used. Breath tests are emerging as a promising, noninvasive diagnostic tool for GC screening and risk stratification [50]; they analyze the metabolic changes associated with GC. Other developing methods include the detection of volatile organic compounds in urine, feces, and sweat. Gas chromatography coupled with mass spectrometry and sensor-based analysis, which distinguishes between disease and control cases using mathematical models based on pattern recognition, are two of the most promising techniques that are being developed.

Another interesting point-of-care testing device is a smartphone-assisted biosensor that is user-friendly, compact, and informative. Early cancer diagnosis with smartphones is based on the detection of molecular cancer biomarkers, including DNA, miRNA, proteins, exosomes, cancer cells, and metabolites. This method is still in the developmental phases [51].

### 4.2. Image-Enhanced Endoscopy

**Figure 2:**
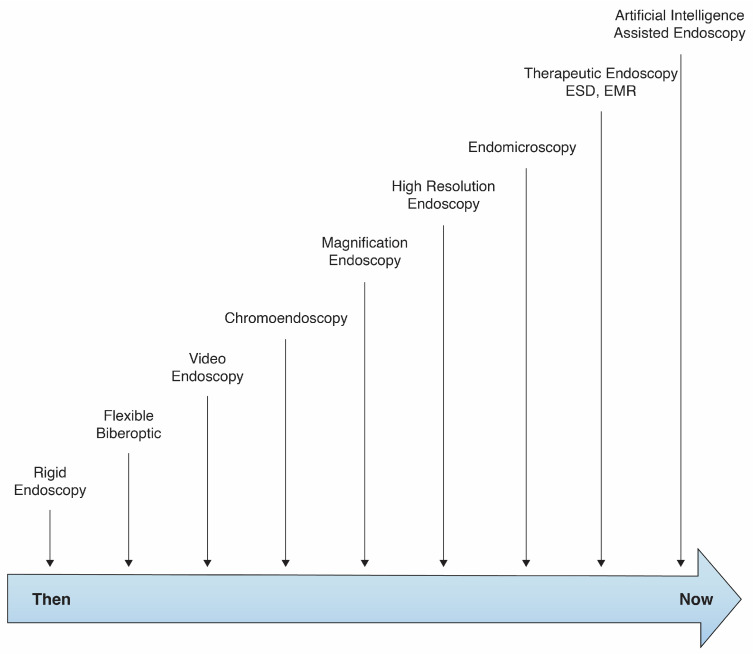
Evolution of endoscopic imaging technologies, from rigid endoscopy to artificial intelligence-assisted systems.

Image-enhanced endoscopy (IEE) plays an important role in cancer management. The early diagnosis of GC leads to better treatment outcomes, and the 5-year survival of EGC often exceeds 90% [52,53]. Conventional white light endoscopy (WLE) has a relatively low sensitivity for detecting EGC (40–60%) and a specificity between 67.9% and 94.3% [54,55], leading to innovations for better visualization [54,56,57]. These innovations include narrow-band imaging (NBI), blue laser imaging (BLI), linked color imaging (LCI), texture/color enhancement imaging (TXI), and electronic tone/texture processing (Pentax i-scan). These techniques improve visualization of lesions by allowing for better characterization of microvascular and microsurface features, leading to better endoscopic resection planning or biopsy [54,56,58,59]. As illustrated in Figure 2, endoscopic technology has evolved from rigid endoscopy to artificial intelligence-assisted systems.

NBI employs narrow bands of blue/green light to improve visualization of capillaries and mucosa, and magnifying endoscopy (ME) NBI improves the visualization of microvascular and microsurface architecture. Diagnostic algorithms such as the Magnifying Endoscopy Simple Diagnostic Algorithm for EGC (MESDA-G), which is a system developed for identification of demarcation lines and false patterns of EGC, have been created to use ME-NBI [60,61,62]. BLI is a laser-based technique operating with dual wavelengths of 410 nm and 450 nm, and it improves vascular and surface contrast. ME-BLI has been evaluated for efficacy in the demarcation of lesions during barrier margin pre-endoscopic submucosal dissection (ESD) evaluation. IEE techniques using electronic post-processing (l-scan) and color-enhancement techniques (LCI/TXI) improve the apparent brightness and color contrast of lesions. This is advantageous for the detection of lesions in atrophic mucosa, where white light imaging offers limited contrast [57,63,64].

A meta-analysis demonstrated that the global sensitivity and specificity for NBI was 87% and 97%, respectively, in the detection of dysplasia or early neoplasia [58]. The use of color-enhancement techniques (LCI/TXI) shows consistent gains in visibility and diagnostic accuracy, particularly for less-experienced endoscopists [65,66,67]. However, there are still challenges, including false negatives in ME-NBI. Diagnosis in certain clinicopathological states is also problematic, leading to follow-up biopsies and further investigations [68]. The most effective approach is to combine wide-field imaging and color-enhancement modes (LCI/TXI/i-scan) for the initial detection, followed by focused magnified inspection, with the aid of NBI or BLI for diagnosis and demarcation of margins. Comparative and observational studies have confirmed this method [59,62,65]. The effective use of these techniques is dependent on endoscopist training. There is evidence that good interobserver agreement and performance can be achieved by inexperienced endoscopists if structured training is given to them [55,67,68].

There are also disadvantages for some IEE modes, including decreased visibility and operator dependence. Future directions must follow the established training modalities. Multicenter comparative trials investigating modes of diagnostic techniques, standard settings, and line algorithms (EGGIM/MESDA-G) [69,70] must be conducted, along with validation studies for artificial intelligence (AI) real-time assessment testing in line with ME-NBI/ME-BLI and wide-field color modes [71,72].

### 4.3. Virtual Chromoendoscopy

Virtual chromoendoscopy (VCE) is a technique that uses optical narrow-band filtering and digital post-processing to enhance the contrast of the mucosal color and vascular patterns using NBI, BLI, and electronic i-scan [60]. VCE promotes the visibility of lesions, aids in vivo optical diagnosis, and assists delineation of margins prior to ESD. Systematic reviews and performance recommendations of the ESGE suggest the routine application of VCE as an image-enhanced technique and a quality measure in upper endoscopy [20]. A 2020 meta-analysis demonstrated pooled per-biopsy sensitivity of 87% and specificity of 97% for NBI in dysplasia. It found that magnifying inspection supplemented by the vessel-plus-surface (VS) technique improved the characterization [73].

NBI narrows the wavelengths of blue–green light that are used to highlight superficial capillaries, BLI employs two competing laser wavelengths that heighten the contrast of microvascularity, and i-scan utilizes patterns of tone and texture enhancement in real time. These techniques allow magnified observation of microvascularity and microsurface patterns and then VS is applied [58]. Intestinal metaplasia has signs of light blue crest and white opaque substance. EGC has irregularity and destruction of microvascularity and microsurface patterns, and clean definition is noted at the limits of lesions identified on magnifying VCE [74]. The diagnostic yield from high-definition WLE therefore depends on the type of lesion and the operator. Structured training and reporting on quality metrics leads to increased accuracy and interobserver agreement. AI computer-aided diagnosis (CADx) for magnifying VCE has produced encouraging results but requires multicenter prospective validation before routine application [75].

### 4.4. Magnification Endoscopy (ME)

ME (optical zoom using dedicated high-definition magnifying endoscopes) allows for various degrees of optical magnification, from near focus to high magnification (up to 150×), giving rise to ME-IEE in conjunction with image-enhancing modalities (NBI, BLI, LCI, and TXI) for the visualization of gastric microvessels and microsurface structures (10–71 µm) [74,76,77]. ME-IEE aids in the detection, optical characterization, and precise delineation of EGC by establishing a demarcation line and identifying abnormal microvascular patterns and microsurface patterns (MSPs). These parameters provide valuable information about the margins prior to ESD and in the optical staging of GC [78]. The VS classification and the MESDA-G require the existence of a demarcation line and either an abnormal microvascular pattern or MSP and are endorsed by several papers [29,56,79].

ME-IEE has been shown to allow for better characterization of lesions and better delineation of the margins when compared with high-definition examination by WLE and non-magnifying VCE. The reported sensitivity, specificity, accuracy, AUROC, and interobserver agreement vary from series to series but generally favor ME-IEE for its reproducibility enhanced by properly structured training [59,74,80]. Adjuncts such as near focus, topical acetic acid, and water immersion can be used to induce the contrast of the surface for flat or treated mucosae [81,82]. Clinicians should also account for inflammation, the effect of *H. pylori* eradication, or the absence of MSP in some undifferentiated cancers because these effects may mimic neoplasia or hamper its identification [35,36].

### 4.5. Magnetic Capsule

Magnetically controlled capsule endoscopy (MCE) or magnetically controlled capsule gastroscopy utilizes a magnetic field placed externally to indirectly control a swallowable video capsule for operator-led unsedated inspection of the esophagus and stomach [83,84,85]. Commercially produced investigational systems comprise NaviCam (IntroMedic Co., Ltd., Seoul, Republic of Korea) and detachable string-based MCE systems with alternative, second-generation motorized and cable systems under production [85,86].

Various parameters of the device, such as the size of the NaviCam (27.0 mm × 11.8 mm, a visual angle of 140 degrees, and a frame rate of about 2/s in stomach) mode and a period of functional operation of 10–14 h, are effective determinants of diagnostic yield [87,88]. External magnetic-assisted guidance is the comprehensive mapping of the gastric contents without sedation, yielding a high acceptance rate amongst patients. Numerous studies measuring overall feasibility and screening efficacy indicated that MCE provided a useful triage/screening, as well as lesion mapping, tool prior to targeted endoscopy [84,87,88].

Comparative studies showed that detachable string-based MCE was an effective method for detecting focal lesions and varices; however, its operative results for EGC and solitary flat precursor lesions showed discrepancies in sensitivity and specificity. Therefore, high insertional definition endoscopy for histopathological certainty and/or possible interventional treatment is necessary [85,89,90]. Recommended general operational protocols for MCE include fasting, abdominal water distension, simethicone administration, and standardized repositioning of the subject. Contraindications are concordant with those for conventional endoscopy techniques (e.g., implanted electromagnetic devices and severe dysphagia). Limitations of MCE include blind areas due to fluid/bubbles, inability to obtain biopsy samples, barrier to entry, and expense. Future roles of MCE include AI utilization for effective detection to improve the accessibility and efficiency of the procedure [91,92].

### 4.6. Optical Coherence Endoscopy (OCT)

Optical coherence endoscopy covers a range of techniques, including OCT, probe-based OCT, optical coherence endomicroscopy, and volumetric endoscopic OCT. This method measures backscattered near-infrared light. It generates depth-resolved cross-sectional and volumetric maps of mucosal microstructures. These maps are almost identical to histological architecture in vivo [20,47]. Endoscopic applications include circumferential rotating catheters, forward-viewing MEMS-mirror probes, and scanning-fiber (Lissajous or single-fiber) forward scanners. Miniaturized MEMS and scanning-fiber designs allow for compact endoscopic integration with high resolution and high frame-rate volumetric acquisition amenable to real-time mapping [93,94,95].

In GC and dysplasia, OCT visualizes layered mucosal architecture, patterns of pits/glands, and depth-resolved attenuation or stiffness (OCT-elastography), supporting detection, lesion characterization, and submucosal invasion estimation with histological correlation in ex vivo and early in vivo studies. A diagnostic study showed high OCT-based discrimination of gastric tissue (e.g., depth-resolved attenuation + Support Vector Machine (SVM) accuracy of 98.6%) [96,97]. Moreover, AI-augmented OCT has shown high tumor/normal classification in a variety of organs, but prospective head-to-head eye-pairing comparisons with high-definition WLE, MCE, or VCE and robust interobserver reproducibility data are limited [98,99].

Volumetric OCT can help direct the selection of the margin and depth of assessment during ESD without removal of tissue with robotic/servo-driven or handheld scanning instruments. This process must account for motion artifacts but has the advantages of probe field of view, resolution, and speed [100,101]. Limitations of OCT include the absence of physical tissue sampling, an operator learning curve, and motion/surface artifacts. Future directions should focus on AI/machine-learning interpretation, OCT angiography, and co-registered volumetrics to automate margin mapping and biopsy guidance [102,103].

### 4.7. Artificial Intelligence (AI)

The integration of AI and diagnostic endoscopy enhances the detection, characterization, risk stratification, and planning of ESD for GC through computer-aided detection and CADx [104,105,106]. Modern systems employ convolutional neural networks in WLE, ME, and VCE (NBI, BLI, LCI, and i-scan) for lesion delineation and histological prediction [107]. Reported results vary according to the specific task performed (i.e., artificial neural network approaches to noninvasive screening yielded an 86.8% accuracy rate) [108], and large multiclass convolution neural networks and real-time CADx studies yielded high accuracy rates and area-under-the-curve results for EGC detection and classification [105,106,109].

Randomized and external validity studies indicated that AI can improve blind-spot monitoring and increase lesion detection compared with conventional practice. AI performed comparably to experts in some analyses [110,111,112,113]. Implementation limitations include dataset bias, domain shift, limited multicenter external validation, reproducibility, false positives, and the ongoing necessity for biopsy and histology in existing pre-ESD histological discordance [114,115]. Although real-time inference and work-flow/hardware integration are achievable in existing systems, formal validation in related latency and clinical outcome, prospective multicenter studies, and guideline deployment are needed.

### 4.8. Prevention of GC by Therapeutic Endoscopy

Endoscopic resection (ER) is an established standard method for managing early-stage gastrointestinal tract carcinomas. When utilizing ER as a curative approach, the estimated risk of lymph node metastasis associated with the lesion must be considered. Over the past two decades, ESD has emerged as the preferred treatment for EGCs [116,117]. Reported en bloc and R0 resection rates exceed 90% [118,119]. Large-scale studies have demonstrated that the technique is safe [120,121] and provides high curative resection rates, along with excellent overall and recurrence-free survival outcomes.

According to the 2025 MAPS III guidelines, ESD is recommended for differentiated gastric lesions clinically staged as dysplastic (low-grade or high-grade) or as intramucosal carcinoma regardless of size if non-ulcerated or up to 30 mm if ulcerated. Endoscopic mucosal resection (EMR) may be considered as an alternative for Paris 0-IIa lesions measuring ≤ 10 mm and with a low likelihood of malignancy. Furthermore, the guidelines suggest that ESD can also be considered for malignant lesions with minimal submucosal invasion when differentiated and ≤30 mm or for intramucosal undifferentiated lesions ≤ 20 mm in size if there are no ulcerative findings [20].

According to the American Society for Gastrointestinal Endoscopy (ASGE) guidelines, the selection between EMR and ESD in patients with early-stage gastric adenocarcinoma (GAC) depends on four main factors: (1) the degree of differentiation (well-differentiated or moderately differentiated vs. poorly differentiated); (2) lesion morphology (ulcerated vs. non-ulcerated); (3) histologic type (intestinal vs. diffuse); and (4) tumor size. The ASGE suggests that either ESD or EMR may be appropriate for well-differentiated or moderately differentiated, non-ulcerated, intestinal-type early GACs measuring < 20 mm. However, for lesions between 20 and 30 mm in size with or without ulceration, ESD is preferred over EMR in well-differentiated or moderately differentiated, intestinal-type early GACs. The ASGE advises against surgical resection in cases of early GAC that meet all of the following criteria: well-differentiated or moderately differentiated, intestinal-type histology, and tumor size ≤ 3 cm [122].

EMR was introduced in Japan in 1984 as a procedure involving submucosal injection of saline into the lesion followed by excision with a snare. EMR is an easy-to-perform procedure with few complications and can be applied when the lesion size is small. For lesions smaller than 1 cm, the complete resection rate is 60% when conventional EMR methods are used, whereas the complete resection rate is 20–30% for lesions larger than 2 cm [123,124]. The EMR technique using ligation and EMR with a cap-fitted panendoscope are two modified EMR techniques developed to increase the complete resection rate and facilitate the removal of difficult lesions. Of all the techniques, EMR with circumferential precutting was the most effective method for resecting larger lesions in one piece. Complete resection by EMR cannot be achieved at once in lesions > 2 cm, and piecemeal resection may increase the risk of local recurrence and lead to inadequate histologic staging [125].

ESD was first introduced in 1999 and has been used to remove large lesions en bloc, as well as precancerous gastric lesions. The ESD technique has been widely adopted for the removal of tumors > 2 cm in diameter, demonstrating acceptable complication rates and superior en bloc and complete resection rates compared with conventional EMR. In this technique, the submucosal layer is precisely dissected using through-the-scope endoscopic knives [116,125]. The following steps are required for ESD: inspection and identification of the lesion; circumferential marking of the lesion; submucosal saline injection into the lesion; mucosal incision; and submucosal dissection using a knife [123].

#### 4.8.1. EMR vs. ESD

The EMR method is an easy-to-perform procedure with fewer complications, and it yields good results for small-sized lesions. The rate of complete resection exceeds 50% for lesions measuring under 1 cm. However, patients who underwent ESD had a higher incidence of en bloc resection, complete resection, and curative resection compared with those who underwent EMR. There was no significant difference in the risk of bleeding between ESD and EMR in a meta-analysis by Tao et al. [124,125]. Another meta-analysis from 2024 showed that ESD had significantly higher en bloc resection rates [risk ratio (RR) = 1.51; 95%CI: 1.20–1.91; *p* < 0.00001; I^2^ = 99%], longer procedure durations (RR = 1.20; 95%CI: 0.43–1.97; *p* < 0.00001; I^2^ = 98%), and a lower recurrence rate (RR = 0.28; 95%CI: 0.14–0.57; *p* = 0.0004; I^2^ = 43%). The same meta-analysis demonstrated that ESD was associated with higher complete and curative resection rates compared with EMR. According to another meta-analysis, ESD was associated with significantly higher rates of postoperative bleeding and perforation than EMR [126].

Multiple modified techniques have been developed to address the challenges associated with performing conventional ESD. These methods include pocket ESD, modified pocket ESD, bridging ESD, multiple tunnel ESD, hybrid ESD, and traction-assisted ESD [127,128]. Pocket ESD is a well-known adaptation of ESD that involves creating a tunnel beneath the lesion akin to placing a hand inside a pocket. A prospective trial demonstrated that pocket ESD provided a safe and effective option for EGC treatment, improved resection speed, and reduced operation time relative to conventional ESD while maintaining a comparable safety profile [129]. A systematic review included randomized controlled trials comparing different techniques for ESD, including the tunnel/pocket method, traction method, and conventional method. According to sensitivity and subgroup analyses, the traction ESD outperformed other techniques in en bloc resection and procedure duration, whereas tunnel/pocket ESD achieved better results in curative resection and had fewer adverse events [130].

#### 4.8.2. Adverse Events

Bleeding is the most common complication of ER and can be classified as either immediate or delayed bleeding. Immediate bleeding is defined as intraoperative and requires blood transfusion, emergency surgery, or vasopressor therapy. The main risk factors for bleeding are tumor location (more often in the upper and middle thirds) and tumor size > 2 cm [124]. Delayed bleeding has been linked to several factors, including tumor location, larger lesion size (>40 mm), recurrence, the presence of ulcers, advanced patient age (≥80 years), prolonged procedure duration, chronic kidney disease, liver cirrhosis, and the use of antithrombotic medications. Studies have shown that delayed bleeding occurs more often following ESD for lesions located in the lower and middle thirds of the stomach compared with those in the upper third. Recently, bleeding was reported in 7.8% of all cases [116].

Perforation during ER ranges from 1.2% to 9.6% but can often be managed conservatively through complete endoscopic closure using endoclips. Delayed perforation because of artificial ulcers following ER is reported in 0.06–0.45% cases, especially after gastric ER [127]. Perforations associated with ESD are generally categorized as macroperforations and microperforations. The underlying mechanism of delayed perforation involves thermal injury from electrical cautery during submucosal dissection or from repeated coagulation. This leads to ischemic damage of the gastric wall and subsequent necrosis [124]. A mucosal defect involving more than 75% of the luminal circumference or extending longitudinally beyond 5 cm has been associated with post-ER stenosis. This is a particular concern following resections in the cardia and pyloric regions. The reported incidence of gastric stenosis after ER ranges from 0.7% to 1.9%. It has been shown that that ER procedures carry a moderate risk of venous thromboembolism. Patients who developed deep vein thrombosis (DVT) exhibited higher D-dimer levels compared with those without DVT. The post-ESD D-dimer values, especially those measured on the day after the procedure, potentially correlate with the presence of DVT [124].

## 5. Conclusions

Despite the decreasing incidence of GC, it is still an important pathology. Intestinal-type GC is a preventable disease. The primary prevention includes the eradication of *H. pylori* and a diet low in salt, no smoking, avoiding smoked and preserved food, and increasing vegetable and fruit intake. Secondary prevention includes the development of markers of atrophy, such as liquid biopsy serological biopsy, and the evolution of endoscopy to detect preneoplastic lesions. Advancements in endoscopic treatment (EMR and ESD) have led to improved outcomes for patients with EGC.

## Figures and Tables

**Table 1:** The ABC method for detection of gastric atrophy [46].

Group	A	B	C	D
Pepsinogens	Normal	Normal	Decreased	Decreased
*H. pylori*	Absent	Present	Present	Absent

## Data Availability

No new data were created or analyzed in this study.

## Abbreviations

The following abbreviations are used in this manuscript:

GC	Gastric cancer
*H. pylori*	Helicobacter pylori
MAPS	Management of epithelial precancerous conditions and early neoplasia of the stomach
EGC	Early gastric cancer
Cag A	Cytotoxin-associated gene A protein, usually written as CagA
PG	Pepsinogen(s)
PGI	Pepsinogen I
PGII	Pepsinogen II
ABC method	Serologic risk stratification using *H. pylori* status plus pepsinogens
ESGE	European Society of Gastrointestinal Endoscopy
MAPS III	MAPS guideline, version III
RNA	Ribonucleic acid
mRNA	Messenger RNA
miRNA	MicroRNA
DNA	Deoxyribonucleic acid
OCT	Optical coherence tomography
MEMS	Microelectromechanical systems
SVM	Support Vector Machine
WLE	White light endoscopy
IEE	Image-enhanced endoscopy
NBI	Narrow-band imaging
BLI	Blue laser imaging
LCI	Linked color imaging
TXI	Texture and color enhancement imaging
ME	Magnifying endoscopy
MESDA-G	Magnifying Endoscopy Simple Diagnostic Algorithm for early Gastric cancer
ME-NBI	Magnifying endoscopy with narrow-band imaging
ME-BLI	Magnifying endoscopy with blue laser imaging
VS	Vessel-plus-surface classification or technique
i-scan	Pentax electronic image enhancement
EGGIM	Endoscopic grading of gastric intestinal metaplasia
AI	Artificial intelligence
CADx	Computer-aided diagnosis
MCE	Magnetically controlled capsule endoscopy or capsule gastroscopy
AUROC	Area under the receiver operating characteristic curve
ER	Endoscopic resection
ESD	Endoscopic submucosal dissection
R0	Microscopically margin-negative resection
EMR	Endoscopic mucosal resection
ASGE	American Society for Gastrointestinal Endoscopy
GAC	Gastric adenocarcinoma
RR	Risk ratio
CI	Confidence interval
I^2^	I-squared heterogeneity statistic
DVT	Deep vein thrombosis
